# Out-of-Reference Range Thyroid-Stimulating Hormone Levels in Levothyroxine-Treated Primary Hypothyroid Patients: A Multicenter Observational Study

**DOI:** 10.3389/fendo.2017.00215

**Published:** 2017-09-12

**Authors:** Dilek Gogas Yavuz, Dilek Yazıcı, Lezzan Keskin, Ayşegül Atmaca, Seda Sancak, Fulden Saraç, İbrahim Şahin, Oğuz Dikbaş, Zeliha Hekimsoy, Serap Yalın, Melin Uygur, Murat Yılmaz, Sibel Yirmibeşcik, Özlem Asmaz

**Affiliations:** ^1^Marmara University, Istanbul, Turkey; ^2^Malatya Devlet Hastanesi, Malatya, Turkey; ^3^Ondokuz Mayıs University, Samsun, Turkey; ^4^Fatih Sultan Mehmet Eǧitim Ve Araştırma Hastanesi, Istanbul, Turkey; ^5^Ege University, Izmir, Turkey; ^6^İnönü University, Malatya, Turkey; ^7^Abant İzzet Baysal University, Düzce, Turkey; ^8^Celal Bayar University, Manisa, Turkey; ^9^Giresun Devlet Hastanesi, Giresun, Turkey; ^10^Namık Kemal University, Tekirdağ, Turkey; ^11^Üsküdar Devlet Hastanesi, Istanbul, Turkey; ^12^Kilis Devlet Hastanesi, Gaziantep, Turkey

**Keywords:** hypothyroidism, levothyroxine, target thyroid-stimulating hormone, thyroid-stimulating hormone, primary hypothyroidism, compliance

## Abstract

**Objective:**

Although levothyroxine (LT4) replacement therapy for hypothyroidism has been established as safe, inexpensive and effective, many studies from different countries reported out-of-reference range thyroid-stimulating hormone (TSH) values for the hypothyroid patients under LT4 treatment. The aim of this study was to determine TSH levels of primary hypothyroid patients under LT4 treatment and to assess self-reported compliance with daily LT4 intake in tertiary care centers in Turkey.

**Design:**

In this cross-sectional, observational study, adult patients with primary hypothyroidism, receiving LT4 treatment for at least 6 months, were included. The patients were from 12 tertiary care centers in 9 cities of Turkey. TSH and free T4 levels were recorded from patient files and self-reported compliance with daily LT4 intake was assessed by interviewing the subjects at the last visit.

**Results:**

A total of 1,755 subjects (46 ± 13 years; F/M: 89.9/10.1%) with primary hypothyroidism were enrolled. Of the hypothyroid subjects, 44.8% had out-of-reference range serum TSH levels. TSH values were over the reference range (TSH > 4 mIU/L) in 26.2% and were under the reference range (TSH < 0.5 mIU/L) in 18.6% of the patients. Total duration of LT4 treatment was 5.9 ± 4.7 years and mean dose was 1.2 ± 0.6 μg/kg/day. Non-compliant patients (31.1%) had higher TSH levels (6.9 ± 16 vs 3.8 ± 0.9 mIU/L, *P* = 0.01) compared to compliant patients.

**Conclusion:**

The results of this study revealed that nearly half of the hypothyroid patients had out-of-reference range serum TSH values, despite under LT4 treatment. Compliance with LT4 treatment seems to be one of the major determinants to reach the target TSH levels in hypothyroid patients.

## Introduction

Primary hypothyroidism is one of the most common endocrine disorders that affects women more frequently and its incidence increases with age. Prevalence of hypothyroidism in the general population ranges from 3.8 to 4.6% (1–4).

Lifelong replacement therapy with levothyroxine (LT4) is usually the treatment of choice for hypothyroidism. Although LT4 therapy has been established as a safe, cheap, and effective therapy, many studies continue to show that LT4 therapy is not always efficacious in achieving normal thyroid-stimulating hormone (TSH) values in hypothyroid patients (2, 4).

Clinical problems related to TSH concentration need to be clarified in patients under LT4 therapy. An observational study by Flynn et al. suggested that increased serum TSH (>4 mU/L) or suppressed TSH (<0.03 mU/L) levels put the patient at risk for cardiovascular diseases, dysrhythmias, osteoporosis, and fractures (5).

Many patients under LT4 therapy are either over-treated or under-treated due to drug interactions, malabsorption syndromes, pancreatic and liver disorders, autoimmune gastritis, high fiber diet, and more frequently non-compliance with LT4 therapy. Factors such as age, etiology of hypothyroidism, concomitant medications, and concomitant illnesses, etc. are also known to alter serum TSH levels, stressing the need for individualization of dosage (6).

Non-compliance with medication may be attributed to the inconveniences and limitations imposed by the therapy: the need to take the medication on a daily basis while fasting, the need to wait approximately 30 min for the next meal and the need to avoid other medications that may interfere with absorption such as calcium carbonate, ferrous sulfate, bile acid sequestrants, sucralfate, and some other drugs may interfere with LT4 metabolism, such as proton pump inhibitors, anticonvulsants, carbamazepine, phenytoin, and phenobarbital (7).

There is a lack of data on the TSH levels of Turkish hypothyroid patients. This study was undertaken to assess the TSH levels in a population of Turkish patients with primary hypothyroidism and to determine the percentage of TSH levels that are out-of-reference range. A second aim was to establish the etiology of out-of-reference range serum TSH values, like the presence of confounding factors such as age, etiology of hypothyroidism, concomitant medication, illness, failure to adjust dose for such confounders, and self-reported compliance with LT4 treatment.

## Materials and Methods

This was an observational, cross-sectional out-patient study conducted at 12 medical centers located in 9 cities of Turkey. The study was approved by the Local Ethics Committee of Marmara University School of Medicine, Istanbul, and was carried out in accordance with the Declaration of Helsinki. All subjects gave informed consent before participation.

### Patient Selection

Adult patients (aged ≥18 years) with primary hypothyroidism under treatment with LT4 for at least 6 months prior to enrollment were included in the study.

Patients with central hypothyroidism, transient hypothyroidism, inflammatory bowel disease, malabsorption syndrome, gastric bypass surgery, a history of celiac disease and pernicious anemia, malignancy or thyroid cancer, and those receiving tri-iodothyronine (T3) therapy, and also pregnant women and lactating mothers were excluded from the study. The patients were especially questioned for the presence of a gastrointestinal disorder that might have caused malabsorption, like the presence of celiac disease or vitamin B12 deficiency.

### Measurements

After medical history was taken, physical examination including height, weight, waist/hip ratio, body mass index (BMI), and blood pressure of the subjects were recorded along with a review of previous medical/laboratory record at the last polyclinic visit.

Latest measured serum TSH and LT4 levels were recorded from the patient files. Serum TSH levels were measured with third-generation immunochemiluminescence method.

Thyroid hormones were measured at the laboratories of the hospitals where the patients were followed. Reference ranges of TSH and FT4 differed between labs.

The dose, duration, brand of LT4 tablet used, concomitant medications, and illnesses were documented. The etiology of hypothyroidism was recorded. Compliance to LT4 therapy and polyclinic visit frequency were assessed by interviewing subjects and by obtaining the information needed from the patient files.

Patient habits of LT4 administration were collected with interview. The questions were about pill brand, ingestion time of the pill, meal time after taking pill, concomitant pill ingestion (proton pump inhibitors, multivitamins, iron, antihypertensive pills, oral antidiabetics, anticonvulsions, etc.), taking pill with water or other liquids (juice, soda, milk, tea, coffee) and taking daily alternate or fixed LT4 dosage.

Self-reported compliance with LT4 therapy was assessed by asking the patients the number of doses missed in the last 1 month and was categorized into three groups (8). The three groups were compliant with treatment: missed <5% dose in the last 1 month, moderately compliant to treatment: missed ≥5%–<15% dose in the last 1 month, and non-compliant to treatment: missed ≥15% dose in the last 1 month.

Based on the current serum TSH levels, whole participants were classified according to the suggestions of American Thyroid Association Guidelines for the treatment of hypothyroidism published in 2014 (9). A value within the reference range for the TSH (0.4–4.0 mIU/L) was considered as the therapeutic target.

Thyroid-stimulating hormone values were classified into over and under replacement. Over-replaced patients were patients with TSH < 0.4 mIU/L and under-replaced patients were patients with TSH value above 4 mIU/L.

### Statistical Analysis

Continuous variables were summarized using descriptive statistics presented as mean and SD. Categorical variables were summarized using counts and percentages. The counts and percentage of subjects who had serum TSH values higher than the normal range were calculated along with two-sided 99% CI. Percentage of subjects in various adherence categories was calculated and any implication of poor compliance with serum TSH levels was evaluated using Chi-square test and logistic regression.

Subgroup analysis was undertaken in a *post hoc* fashion to assess the impact of possible confounding factors (age, gender, etiology of primary hypothyroidism, etc.) on serum TSH levels. For such subgroup analysis, serum TSH levels were compared using analysis of variance or Kruskal–Wallis test as applicable. Pearson correlation with two-tailed probability values was used to estimate the strength of association between variables.

A multiple linear regression analysis was performed to define the predictors of serum TSH levels. Predictors with possible influence on dependent variable were added as covariates (being compliant, age, duration of treatment, BMI, daily LT4 dosage).

All statistical calculations were performed with GraphPad InStat software for Windows (San Diego, CA, USA). The level of statistical significance was set at *P* < 0.05.

## Results

A total of 1,755 subjects were enrolled in the study across 9 cities from 12 medical centers in Turkey. Out of the 1,755 subjects, 1,578 (89.9%) were female and 177 (10.1%) male. Demographic and clinical parameters, serum TSH and free T4 levels of the study group are shown in Table 1.

**Table 1:** Demographic characteristics, clinical, and laboratory parameters of the study group.

	Total group (*n*: 1,755)	Women (*n*: 1,578)	Men (*n*: 177)	*P*
Age (years)	46.9 ± 13.2	46 ± 12	49 ± 0.9	n.s.
BMI (kg/m^2^)	28.9 ± 6.1	29.0 ± 5.9	28.5 ± 7.1	n.s.
Duration of disease (years)	5.83 ± 5.1	5.9 ± 4.5	5.1 ± 4.2	n.s.
LT4 daily dosage (μg/day)	96.0 ± 47.9	94.9 ± 47	103.7 ± 49	n.s.
LT4 dosage per kg (μg/kg)	1.28 ± 0.64	1.27 ± 0.62	1.36 ± 0.7	0.001
fT4 (ng/dL)	1.24 ± 0.37	1.24 ± 0.36	1.29 ± 0.41	n.s.
TSH (mIU/L)	4.6 ± 11	4.5 ± 10	5.2 ± 17	n.s.
<0.5 mIU/L, *n* (%)	327 (18.6)	280 (17.7)	47 (26.5)	n.s.
0.5–4 mIU/L, *n* (%)	950 (54.1)	874 (55.3)	76 (42.9)	n.s.
4–10 mIU/L, *n* (%)	337 (19.2)	303 (19.2)	34 (19.2)	n.s.
>10 mIU/L, *n* (%)	141 (8.1)	121 (7.6)	20 (11.2)	0.01

*BMI, body mass index; LT4, levothyroxine; fT4, free T4; TSH, thyroid-stimulating hormone*.

Mean TSH level was 4.6 mIU/L in the whole group. Population distribution according to TSH levels is shown in Table 1. Of the patients, 44.8% had out-of-reference range serum TSH levels. In whole study group, 26.2% (*n*: 478) of the patients were under-replaced (TSH > 4 mIU/L) and 18.6% (*n*: 327) of the patients were over replaced (TSH < 0.5 mIU/L).

Thyroid-stimulating hormone levels and LT4 dosage according to age are shown in Table 2. In patients younger than 65 years of age (*n* = 1,659), target TSH level of 0.4–4 mIU/L was observed in 37.9% (*n* = 630) of the patients, 18.3% (*n* = 304) of the patients had TSH levels <0.5 mIU/L. In patients older than 65 years of age (*n* = 95), mean TSH level was 5.7 ± 13 mIU/L. In this group, 32.6% (*n* = 31) of the patients had TSH levels between 0.5 and 4 mIU/mL, 25.2% (*n* = 24) had TSH levels <0.5 mIU/L.

**Table 2:** TSH values and LT4 dosage according to age.

	18–29 (*n*: 188)	30–39 (*n*: 320)	40–49 (*n*: 455)	50–59 (*n*: 478)	60–69 (*n*: 248)	70–79 (*n*: 54)	80–89 (*n*: 12)	*P*
TSH (mIU/mL)	5.5 ± 2.8	5.2 ± 3.5	5.1 ± 3	3.9 ± 0.4	3.03 ± 2	6.2 ± 2.1	4.8 ± 5.2	0.04
Duration of treatment (yrs)	5 ± 3.7	4.6 ± 4	5.7 ± 4	6.2 ± 5.6	7.3 ± 6	8.2 ± 5.2	13.7 ± 9	0.04
Daily dose (µg/day)	95.4 ± 49	95.7 ± 45	104 ± 56	91.3 ± 43	87.9 ± 39	94.9 ± 42	73.8 ± 35	0.001
Daily LT4 dosage (µg/kg)	1.44 ± 0.8	1.51 ± 0.6	1.34 ± 0.6	1.72 ± 0.3	1.59 ± 0.2	1.24 ± 0.5	1.04 ± 0.4	0.001

*Yrs, years; LT4, levothyroxine*.

### Analysis of Subjects according to the Etiology of Primary Hypothyroidism

Etiology of hypothyroidism was defined as chronic autoimmune thyroiditis or atrophic thyroiditis (63.6%), total thyroidectomy for multinodular goiter (33.5%), and radioactive iodine (RAI) ablation therapy for Graves disease or toxic nodular goiter (2.8%) (Table 3). Clinical and laboratory parameters according to etiology are shown in Table 3. Patients with autoimmune thyroiditis had lower daily dose of LT4 and shorter duration of treatment compared to patients with postsurgical and RAI ablation therapy for hyperthyroidism group.

**Table 3:** Demographic and labororatory parameters of the study groups according to the etiology of primary hypothyrodism.

	Autoimmune (*n* = 1,117)	Postsurgical (*n* = 588)	Post radioactive ablation (*n* = 50)	*P*
Age (yrs)	45.4 ± 13.2	50.2 ± 11.6	52.5 ± 10	n.s.
Gender (M/F)	101/1,016	65/523	11/39	n.s.
Duration of disease (yrs)	4.99 ± 5.8	7.05 ± 6.9	7.15 ± 6.5	<0.0001
Daily dose of LT4 (μg/day)	88.1 ± 45.3	112.6 ± 47.7	109.7 ± 52.4	<0.0001
Daily dose of LT4 (μg/kg)	1.18 ± 0.57	1.49 ± 0.7	1.47 ± 0.83	<0.001
TSH (mIU/L)	4.53 ± 11.4	6.03 ± 14.7	2.93 ± 3.3	<0.0001
fT4 (ng/dL)	1.22 ± 0.32	1.28 ± 0.4	1.40 ± 0.40	<0.0001

*Yrs, years; M, male; F, female; LT4, levothyroxine; fT4, free T4; TSH, thyroid-stimulating hormone*.

### Analysis of Subjects according to LT4 Ingestion Patterns

In terms of missed doses, 550 (31.3%) patients reported that they missed more than one dose in a month (Table 4). Self-reported missed LT4 doses ranged between 1 and 30 and mean ± SD for missed doses was 4.3 ± 6.1 for the last month during the interview.

**Table 4:** LT4 ingestion patterns of the subjects.

	*N* (%)
**LT4 dosage**	
Fixed	1,135 (64.6)
Alternate	620 (35.4)
**Compliance**	
Non-compliant	550 (31.3)
Compliant	1,205 (68.7)
**Time for medication**	
>60 min before breakfast	390 (22.2)
30–60 min before breakfast	782 (44.5)
<30 min before breakfast	543 (30.9)
After breakfast	2 (0.1)
No answer	38 (2.1)
**LT4 ingestion**	
Alone	1,269 (72.3)
With other medications	486 (27.7)

*LT4, levothyroxine; min, minutes*.

Most of the people were taking the medication between 30 and 60 min before breakfast (*n*: 782, 44.5%). There was no difference between the TSH levels of the patients taking the medication 30–60 min before breakfast and less than 30 min before. Twenty-seven percent (*n* = 486) of the patients reported taking LT4 pills together with other pills (proton pump inhibitors, multivitamins, iron, antihypertensive pills, oral antidiabetics, etc.). Their TSH levels were not different than the ones who were taking the LT4 pill alone. The only available preparation for LT4 is in the tablet form and some pill dosages are not in the market in Turkey. Patients were sometimes recommended by physicians to take alternate daily doses to adjust their LT4 requirements. The percentage of patients taking alternate LT4 dosage was 35.4% (*n* = 620). TSH levels were similar between patients taking alternate and fixed doses. Daily LT4 dosage was higher in patients using alternate daily dosage (99.2 ± 49.4 µg/day) compared to fixed daily dosage (94.0 ± 47 µg/day) (*P* < 0.001) (Table 5).

**Table 5:** Analysis of subjects according to taking fixed and alternating doses.

	Fixed dosing (*n* = 1,135) (%64.6)	Alternate dosing (*n* = 620) (%35.4)	*P*
Age (yrs)	46.8 ± 13.2	46.9 ± 13.2	n.s.
Duration of LT4 usage (yrs)	5.6 ± 6.8	6.2 ± 6.7	n.s.
Daily dose of LT4 (μg/day)	94.0 ± 47	99.2 ± 49.4	<0.001
Daily dose of LT4 (mg/kg)	1.57 ± 5.5	1.31 ± 1.4	n.s.
TSH (mIU/L)	5.02 ± 11	3.9 ± 12	0.059
fT4 (ng/dL)	1.93 ± 7.8	2.2 ± 8.6	n.s.

*Yrs, years; LT4, levothyroxine; fT4, free T4; TSH, thyroid-stimulating hormone*.

### Analysis of Subjects according to Being Compliant and Non-compliant

Mean TSH levels were 6.76 ± 15.2 mIU/L and 3.65 ± 9.0 mIU/L in non-compliant (*n*: 550, 31.3%) and compliant groups (*n*: 1,205, 68.7%), respectively (*P* = 0.01) (Table 6). There was no difference between the compliant and non-compliant group in terms of etiology, LT4 daily dosage, age, and duration of disease. Patients with TSH > 10 mIU/L (*n*: 134) had higher non-compliance rate (56.7%) comparing to the whole study group (31.3%) (*P* < 0.01).

**Table 6:** Analysis of subjects according to being compliant and non-compliant.

	Non-compliant (*n* = 550) (31.3%)	Compliant (*n* = 1,205) (68.7%)	*P*
Age (yrs)	44.5 ± 13.5	48.0 ± 12.9	n.s.
Duration of disease (yrs)	5.7 ± 7.0	5.9 ± 6.6	n.s.
Daily dose of LT4 (μg/day)	96.6 ± 47.7	95.5 ± 48.2	n.s.
Daily dose of LT4 (μg/kg)	1.30 ± 0.66	1.31 ± 1.4	n.s.
TSH (mIU/L)	6.76 ± 15.2	3.65 ± 9.0	0.01
fT4 (ng/dL)	1.77 ± 6.3	2.17 ± 8.8	n.s.

*Yrs, years; LT4, levothyroxine; fT4, free T4; TSH, thyroid-stimulating hormone*.

### Correlation Analysis

There was an inverse correlation between serum TSH and free T4 levels (*r* = −0.43, *P* < 0.0001) in the whole study group. Daily LT4 dosage were positively correlated with BMI (*r* = 0.16, *P* < 0.001) and negatively correlated with serum TSH level (*r* = −0.17, *P* < 0.0001).

### Multiple Regression Analysis

In multiple regression model, independent predictors of serum TSH level were being compliant, age, and daily LT4 dosage (kg/day) (*r*^2^ = 1.84, *P* < 0.0001).

## Discussion

A total of 44.8% of hypothyroid subjects had TSH levels that were out-of-reference range (26.2% being under-treated and 18.6% over-treated). According to our results, the percentage of over and under-replaced patients was more in the non-compliant group, compared with the compliant group. Besides, TSH levels were higher in the non-compliant group when compared with the compliant group.

Considering the etiology, patients with autoimmune etiology required lower amount of LT4 compared to the others, while TSH levels were highest in the group of patients that had postsurgical hypothyroidism.

Thyroid hormone replacement therapy is usually simple and inexpensive, with usually once-daily dosing due to the long half-life of LT4. Once-daily dosing leads to optimal clinical response and TSH levels of these patients are in the normal ranges. The replacement dosage usually remains stable for years. However, about 10% of the patients are not satisfied. Some other patients are not well controlled and need unusually high doses of LT4 (7).

It has been shown that up to 40% of patients were under-treated, and also up to 40% over-treated, especially in the elderly (2, 8–10). A study evaluating elderly hypothyroid patients showed that more than 40% had low and 16% had high TSH levels, which were possibly correlated with lower weight (10). A survey conducted in Colorado revealed that up to 40% of patients taking thyroid medications had TSH levels higher than the normal reference range (2).

Thyroid-stimulating hormone values being out of the normal range could be due to the presence of confounding factors, such as failure to adjust LT4 dose according to the confounders or poor compliance of patients (2, 3, 11, 12). One of the most common reasons for non-compliance is avoiding or forgetting to take the medication since, as most of the chronic diseases, hypothyroidism is a chronic disease, that does not go along with overt symptoms (11, 13).

Regarding the etiology of non-compliance, a study conducted in Brazilian hypothyroid patients revealed that 80% of the patients did not follow physician instructions due to misunderstanding of the prescription or forgetfulness (2, 11).

Levothyroxine has a narrow therapeutic range so precise and consistent dosing is necessary, especially in the elderly patients (14, 15). Using appropriate medication with bioequivalence to the original hormone is very important.

Before considering non-compliance, it is necessary to see if the dose of the medication is adequate. It is especially important to know that certain conditions require higher doses of LT4 replacement, such as pregnancy, childhood, or obesity (15).

The absorption of LT4 occurs mostly within an interval of 3 h from ingestion, mainly in the jejunum and ileum (16, 17). This absorption rate may diminish in the hypothyroid patient (16). Absorption is maximal when the stomach is empty, reflecting the importance of gastric acidity in the process (18, 19). In situations where gastric acid secretion is impaired, the requirement of oral LT4 may increase. This is especially evident in patients with atrophic gastritis and concomitant *Helicobacter pylori* infection (18). Treating *H. pylori* infection decreases the need for LT4 in several populations (18, 20, 21).

Drug interaction may interfere with LT4 absorption (5, 15, 22). In our population, taking the medication with other pills did not seem to affect the final TSH levels. Moreover, there was no difference in terms of TSH levels among patients taking LT4 30–60 min before breakfast compared to taking it less than 30 min before. This pointed out that a decreased absorption time of LT4 may not be such an important aspect affecting TSH levels. Besides, the subjects did not report to have any chronic gastrointestinal diseases that might have caused malabsorption.

Since a great number of studies have demonstrated that timing of LT4 ingestion as well as food and beverages can impair LT4 intestinal absorption, daily administration of LT4 in the morning, on an empty stomach is definitely the best modality (19, 22).

There did not seem to be any difference in terms of the TSH levels between the groups, taking the medication as a fixed dose or as alternate day dosing ın our study. Recent studies in the literature reported that weekly LT4 and daily LT4 supplementation has similar effects on serum TSH levels in hypothyroid patients (21, 23).

Common germline genetic variations may be responsible for the relatively wide range of serum TSH observed in the normal population and may affect the differential bioavailability of thyroid hormones in different tissues (22, 24).

New formulations of LT4 are available in many countries. These new formulations are the oral solution and the soft gel capsule, which due to intrinsic pharmacokinetic characteristics, may solve the problem of tablet LT4 malabsorption in some conditions (25, 26).

The limitation of the study was that although the patients were asked about the history of a disease that might have caused malabsorption, no through investigation, such as endoscopy was performed in this regard. Moreover, with this study, we can only make an assumption about the effect of non-compliance on out-of-reference range TSH levels. It would have been better to take into consideration more than one TSH value, but since this was a cross-sectional study only one value could be obtained. The hormone assays were not the same in different centers but all the laboratories used standardized assays.

In conclusion, a significant percentage of our population with primary hypothyroidism had TSH levels that were out-of-reference range. TSH levels were higher in non-compliant patients compared to the compliant ones. Although drugs and malabsorption syndromes might affect the TSH levels under LT4 treatment, non-compliance may be a factor in determining high TSH levels that were observed in our population of patients.

## Ethics Statement

The study was approved by the local ethics committee of Marmara University School of Medicine and was carried out in accordance with the Declaration of Helsinki. All subjects gave informed consent before participation.

## Author Contributions

DGY, DY, LK, AA, SS, and FS: substantial contributions to the conception or design of the work; or the acquisition, analysis, or interpretation of data for the work; drafting the work or revising it critically for important intellectual content; final approval of the version to be published; and agreement to be accountable for all aspects of the work in ensuring that questions related to the accuracy or integrity of any part of the work are appropriately investigated and resolved. OD, ZH, SY, MU, MY, SYB, OA, and IS substantial contributions to the conception or design of the work; or the acquisition, analysis, or interpretation of data for the work; final approval of the version to be published; and agreement to be accountable for all aspects of the work in ensuring that questions related to the accuracy or integrity of any part of the work are appropriately investigated and resolved.

## Conflict of Interest Statement

The authors declare that the research was conducted in the absence of any commercial or financial relationships that could be construed as a potential conflict of interest.
